# Natural Docosahexaenoic Acid in the Triglyceride Form Attenuates In Vitro Microglial Activation and Ameliorates Autoimmune Encephalomyelitis in Mice

**DOI:** 10.3390/nu9070681

**Published:** 2017-06-30

**Authors:** Pilar Mancera, Blanca Wappenhans, Begoña Cordobilla, Noemí Virgili, Marco Pugliese, Fèlix Rueda, Juan F. Espinosa-Parrilla, Joan C. Domingo

**Affiliations:** 1 Neurotec Pharma SL, Bioincubadora PCB-Santander, Parc Científic de Barcelona, Baldiri Reixac 15, E-08028 Barcelona, Spain; pilar_m_a@hotmail.com (P.M.); blancawappenhans@gmail.com (B.W.); nvt21@hotmail.com (N.V.); marcopugliese@ub.edu (M.P.); juan.espinosa@vhir.org (J.F.E.-P.); 2 Departament de Bioquímica i Biologia Molecular, Falcutat de Biologia, Universitat de Barcelona, Avinguda Diagonal 643, E-08028 Barcelona, Spain; bgcordobilla07@ub.edu (B.C.); frueda@hotmail.es (F.R.); 3 Unitat de Bioquímica i Biologia Molecular, Departament de Ciències Fisiològiques I, Facultat de Medicina, Institut d’Investigacions Biomèdiques August Pi i Sunyer (IDIBAPS) and Centro de Investigación Biomédica en Red sobre Enfermedades Neurodegenerativas (CIBERNED), Casanova 143, E-08036 Barcelona, Spain

**Keywords:** docosahexaenoic acid, microglia, omega-3 polyunsaturated fatty acid, oxidative stress, anti-inflammatory process, EAE model

## Abstract

Many neurodegenerative diseases are associated, at least in part, to an inflammatory process in which microglia plays a major role. The effect of the triglyceride form of the omega-3 polyunsaturated fatty acid docosahexaenoic acid (TG-DHA) was assayed in vitro and in vivo to assess the protective and anti-inflammatory activity of this compound. In the in vitro study, BV-2 microglia cells were previously treated with TG-DHA and then activated with Lipopolysaccharide (LPS) and Interferon-gamma (IFN-γ). TG-DHA treatment protected BV-2 microglia cells from oxidative stress toxicity attenuating NO production and suppressing the induction of inflammatory cytokines. When compared with DHA in the ethyl-ester form, a significant difference in the ability to inhibit NO production in favor of TG-DHA was observed. TG-DHA inhibited significantly splenocyte proliferation but isolated CD4+ lymphocyte proliferation was unaffected. In a mice model of autoimmune encephalomyelitis (EAE), 250 mg/kg/day oral TG-DHA treatment was associated with a significant amelioration of the course and severity of the disease as compared to untreated animals. TG-DHA-treated EAE mice showed a better weight profile, which is a symptom related to a better course of encephalomyelitis. TG-DHA may be a promising therapeutic agent in neuroinflammatory processes and merit to be more extensively studied in human neurodegenerative disorders.

## 1. Introduction

Nerve injury and neuroinflammation can result from a number of physical, chemical, infectious, and immunological causes. Although, in general, microglia cells play a beneficial role in various aspects of maintenance and repair of the central nervous system (CNS), they also contribute to CNS damage through several pathways involved in neuroinflammatory or autoimmune conditions, such as secretion of inflammatory cytokines (e.g., interleukin (IL)-6, tumor necrosis factor (TNF)-α, IL-1) and nitric oxide (NO) [1]. Furthermore, microglia cells in the presence of T-cells are the main factor responsible for the induction of the immune response in brain, where they play a role similar to that of dendritic cells in the periphery in the recognition and presentation of self and foreign antigens via phagocytic activity [2,3]. Non-activated microglia do not express or express low levels of major histocompatibility complex (MHC) class I/II proteins, and therefore act as weak antigen presenting cells (APCs). However, activated microglia cells rapidly express MHC class I/II molecules and become effective APCs. Also, microglia may contribute to the pathology of neurological disorders via non-inflammatory mechanisms, for instance, via signaling pathways that accelerate neuronal apoptosis or by altering amyloid-beta metabolism. When an inflammatory process occurs, T-cells cross the blood-brain barrier, thanks to some specialized surface markers, and are rapidly activated by microglia, which acts as APCs displaying antigens. By these various mechanisms, microglia have been implicated in the progression or adverse outcomes of a number of diseases or injuries, including Alzheimer’s disease (AD), multiple sclerosis (MS), amyotrophic lateral sclerosis (ALS), Parkinson’s disease (PD), stroke, and variant Creutzfeldt-Jakob disease [1,4,5].

Different animal models of neuroinflammation and neurodegeneration have been developed [6,7,8,9,10,11]. Induction of autoimmune encephalomyelitis (EAE) in susceptible strains of mice can be achieved by using proteolipid protein (PLP), myelin basic protein (MBP), myelin oligodendrocyte glycoprotein (MOG), or peptides corresponding to the encephalitogenic portions of these proteins, together with a potent adjuvant and bacterial toxin to induce a strong immunological activation of myelin antigen-specific T-cells and the breakdown of peripheral tolerance.

Mice models of EAE, in spite of some common traits with ALS, have been more frequently associated with MS, since many peculiar pathologies observed in the CNS of mice with EAE bear strong similarities with those found in the CNS of MS patients [12]. Both EAE and MS are demyelinating autoimmune disorders of the CNS mediated by CD4+ T-cell activation associated with demyelinating lesions, which are characterized by mononuclear cell infiltration (T-cells, B-cells, monocytes, macrophages, and neutrophils) due to chemokine production by the pathogenic T-cells [13,14,15]. Activated T-cells express integrins that enable them to cross the blood–brain barrier [16]. In the brain, T-cells are reactivated by microglia cells acting as APCs [17], which with other inflammatory cells leads to the expression of proinflammatory cytokines (Interferon-gamma (IFN-γ), IL-17, Granulocyte Macrophage Colony-Stimulating Factor (GM-CSF), and TNF-α), some of which can directly injure nervous tissue and are largely responsible for destruction of the myelin-sheathed axonal tracts and the development of lesions.

In the EAE model, CNS inflammatory cell infiltration is accompanied by the activation of microglia and the release of proinflammatory cytokines associated with oxidative stress and apoptosis of neuronal tissue [18,19]. The presenting clinical episode of EAE is characterized by an ascending hind limb paralysis beginning in the tail and spreading to the hind limbs and forelimbs. This is considered an acute-phase of the disease and is preceded by pronounced weight loss.

Furthermore, the discovery of the role for activated microglia in all these neuroinflammatory diseases has led to their emergence as a target for therapeutic intervention, either to prevent or treat neuroinflammatory disease such as chronic neurodegenerative disorders, or to improve outcomes after acute episodes or injuries [20,21]. A way of controlling neuroinflammation is by inhibition of microglial activation. Multiples stimuli are able to activate microglia, but it seems that all of them are mainly dependent of the activation of mitogen-activated protein kinase (MAPK) controlled pathways [22]. Accordingly, MAPK-inhibiting drugs may be a promising target for neuroinflammatory diseases.

It has been shown that agonists of peroxisome proliferator-activated receptor-gamma (PPAR-γ) inhibited the production of NO, proinflammatory cytokines (IL-1β, IL-6, and TNF-α), and chemokine monocyte chemotactic protein-1 (MCP-1) in microglia and astrocytes [23]. Oral treatment with pioglitazone, a PPAR-γ agonist, diminished motor neuron loss, enhanced motor performance, delayed weight loss, and also reduced iNOS, NF-κB, and 3-nitrotyrosine levels in spinal cords, prolonging survival by 13% of G93A SOD (transgenic mouse model of ALS) when compared with an untreated control littermate counterparts, suggesting the therapeutic potential of PPAR-γ agonists in human neuroinflammatory diseases [24,25].

In spite of the availability of approved drugs for the treatment of patients with neuroinflammatory disorders, they often have a limited efficacy and a narrow therapeutic index. Thus, there is an urgent need to develop new drugs for the treatment of these patients. Omega-3 fatty acids and especially docosahexaenoic acid (DHA) are natural products which can be taken as dietary supplements, and have been shown to act throughout nuclear PPAR-γ receptors on dendritic cells, modulating their inflammatory activity [26] and converting dendritic cells in poor stimulators of antigen-specific T-cells in terms of proliferation and Th1/Th17 differentiation [3]. Furthermore, these fatty acids have shown modulation of the inflammatory properties of activated microglia [11,27,28,29] and amelioration of neurodegenerative diseases involving neuroinflammation [30,31]. The objective of this study was to determine the capacity of the triglyceride form of natural DHA (TG-DHA), which has shown improved bioavailability [32,33], as compared to the ethyl ester form to modulate the proinflammatory activity of in vitro cultured microglial cells, as well as to assess the effect of TG-DHA in the course of the disease in a mice model of EAE [12].

## 2. Materials and Methods

### 2.1. Reagents

Triglyceride of docosahexanoic acid (TG-DHA) obtained by enzymatic synthesis was kindly supplied by Brudy Technology (Barcelona, Spain). For the ethyl ester of DHA (EE-DHA) Omacor (Pronova BioPharma Norge AS, N-1327 Lysaker, Norway) was used. DHA compounds were prepared in dimethyl sulfoxide (DMSO, Sigma-Aldrich, St. Louis, MO, USA) and diluted in the culture media immediately before cell treatment, being added to the cells at DMSO concentration of 0.5%.

### 2.2. Cell Culture and Treatment

BV-2 cells were cultured in RPMI-1640 medium (Gibco Invitrogen, Paisley, Scotland, UK) supplemented with 10% Fetal Bovine Serum (FBS) and 0.1% penicillin-streptomycin at an initial density of 5 × 10^4^ cells/mL, at 37 °C in a 5% CO_2_. Treatments with TG-DHA (1–20 µM) or EE-DHA (10–20 µM) were performed 30 min before stimulation with Lipopolysaccharide (LPS) 100 ng/mL and IFN-γ 50 pg/mL one day after cells were seeded. Control cells were cultured at the same final concentration of vehicle as DHA-treated cells. Culture supernatants were collected 24 h after LPS/IFN-γ stimulation and stored at −20 °C until assayed for nitrites and TNF-α quantification. Cell viability was determined after treatment with the 3-(4,5-dimethyl-2-thiazolyl)-2,5-diphenyl-2H-tetrazolium bromide (MTT) reduction method.

### 2.3. Nitrite and Cytokine Quantification

Nitrite levels were quantified by the Griess reaction [34]. Briefly, 25 μL of Griess reagent A (sulfanilamide) and 25 μL of reagent B (*N*-1-naphthylethylene-diamine) were mixed with 50 μL of culture supernatants in a 96-well plate. After 10 min at 23–25 °C, color development was measured at 540 nm on a microplate reader (BioTek ELX800, BioTek Instruments Inc., Winoosky, VT, USA). A sodium nitrite standard curve was used to determine the nitrite concentration. IL-6 released into the culture medium was quantified using an Enzyme-Linked Immuno Sorbent Assay (ELISA) kit specific for mouse IL-6 (Mouse IL-6 Ready-SET-Go!^®^, eBioscience, San Diego, CA, USA) and TNF-α with a specific murine TNF-α ELISA kit (Murine TNF-α ELISA Development Kit, Peprotech, Rocky Hill, NJ, USA) following the manufacturer’s instructions.

### 2.4. Cell Viability: 3-(4,5-dimethyl-2-thiazolyl)-2,5-diphenyl-2H-tetrazolium bromide (MTT) Reduction Method

Cell viability was established by MTT reduction assay. Briefly, 96 microplate wells were filled with MTT (Sigma-Aldrich) at a final concentration of 0.5 mg/mL per well and incubated at 37 °C, then DMSO was added and cells were gently resuspended. Absorbance was read at 560 and 620 in a microplate reader (BioTek ELX800, BioTek Instruments Inc., Winoosky, VT, USA).

### 2.5. Mice

Female C57BL/6J mice (8 to 10 weeks old) purchased from Charles River (Sulzfeld, Germany) were handled according to European legislation (86/609/EEC) as previously described [34]. All the experiments and procedures were approved by the Ethics Committee of the University of Barcelona under the supervision of the Generalitat of Catalunya, Spain.

### 2.6. EAE Induction and Treatment

EAE was induced by immunization with >95% pure synthetic MOG35-55 peptide (rat MOG35-55, MEVGWYRSPFSRVVHLYRNGK, EspiKem Srl, Florence, Italy). Mice were injected subcutaneously at one side of the flank with 100 μL solution containing 150 μg of rat MOG in complete Freund’s adjuvant (Sigma-Aldrich) and 5 mg/mL *Mycobacterium tuberculosis* H37Ra (Difco Laboratories, Detroit, MI, USA). Forty-eight hours after MOG injection, 150 ng pertussis toxin (Sigma-Aldrich) in 100 μL PBS were injected intraperitoneally. The animals were monitored every other day for the development of clinical symptoms, which typically become evident between 12 and 20 days after immunization. Once the disease had developed, mice were assessed daily for changes in severity. Signs of EAE were scored daily on a 0 to 6 scale using the following criteria: 0, no clinical signs; 1, distal limp tail; 1.5, complete limp tail; 2, mild paraparesis of the hind limbs, unsteady gait, and impairment of righting reflex; 3, moderate paraparesis, partial hind limb paralysis, voluntary movements still possible, and ataxia; 4, paraplegia and forelimb weakness; 5, tetraparesis; 6, moribund state. Mice were treated daily by oral administration of 50 mg/kg TG-DHA, 250 mg/kg TG-DHA, or vehicle (0.3% DMSO in water) over a total of 56 days, with treatment beginning 15 days prior to EAE induction. At this time mice were euthanized under deep anesthesia to obtain blood and tissues to perform analysis described below. No treatment-related adverse effects were observed in any of either healthy or EAE-induced mice following standard criteria [35]. Experiments were performed with groups (*n* = 10) of C57BL/6J healthy untreated mice (healthy untreated group), healthy DHA-treated mice (healthy DHA-treated group), EAE-induced untreated mice (EAE untreated group), and EAE-induced DHA-treated mice (EAE-DHA-treated group) as indicated. The groups of mice were randomized. No previous selection was made for any characteristics, such as weight, size, etc.

### 2.7. Mouse Splenocytes and CD4+ T-Cells Preparation

Splenocytes were isolated from the spleens of female C57BL/6J mice. Cells were flushed out from the spleens gently using a syringe plunger with PBS and then centrifuged (×1000 rpm, 4 °C). The pellet was resuspended and left 5 min on ice in ACK lysing buffer (Invitrogen, Eugene, OR, USA) to remove erythrocytes. After further centrifugations, cells were resuspended in 10 mL of complete medium RPMI (Invitrogen) supplemented with 10% Fetal Bovine Serum (FBS, Invitrogen), Hepes 10 mM (Invitrogen), sodium pyruvate 1 mM (Invitrogen), non-essential amino acids 1% (Sigma-Aldrich), and 50 µM β-mercaptoethanol (Sigma-Aldrich).

CD4+ T-cells were isolated using Dynabeads FlowComp Mouse CD4 kit (DYNAL, Invitrogen) following the manufacturer’s instructions. After isolation, cells were counted and the viability was determined by trypan blue exclusion, while the purity for CD4+ cells (>92%) was assessed by fluorescence activated cell sorting (FACS) (Gallios BD Bioscience).

### 2.8. Proliferative Response of Lymphocytes

Proliferation assay was carried out by plating 2 × 10^5^ cells/well in 96-well plates followed by exposure to stimulus anti-CD3 (KT3, coated at 1 µg/mL; Serotec, Oxford, England, UK) and anti-CD28 (37.51, coated at 2.5 µg/mL; eBioscience, San Diego, CA, USA) for 72 h. Cell proliferation was determined by colorimetric bromodeoxyuridine (BrdU) cell proliferation kit (Calbiochem, Darmstadt, Germany) following the manufacturer’s instructions. In brief, 20 µL of BrdU labelling solution diluted 1:2000 in a culture medium was added to each well for the last 18 h of culture. After removing the medium, cells were fixed, and the anti-BrdU peroxidase working solution was added to each well and incubated for 1 h at room temperature. Following several washes, the substrate solution was added, color was developed, and absorbance was measured with a microplate reader at 450 nm. Proliferation rate was calculated as the absorbance of cells in the presence of anti-CD3-CD28 divided by the absorbance of cells in the absence of antibodies.

### 2.9. Determination of Tissue Fatty Acids Profile

To check absorption of fatty acids by mice, the fatty acid composition of plasma lipids was assessed by gas chromatography. Plasma total lipids were extracted by the method of Bligh and Dyer with chloroform: methanol (2:1, v:v). Methyl esters of fatty acids were obtained by transesterification with boron trifluoride in methanol at 80 °C for 60 min. An aliquot containing the methyl esters of fatty acids was analyzed by gas chromatography (Hewlett-Packard model 5890) on a 30 m RTX-2330 column (Restek, Bellefonte, PA, USA) with an internal diameter of 0.25 mm equipped with a flame ionization detector. The carrier gas was helium at a pressure of 105 kPa. For the total separation of the different compounds, two temperature settings were established: 140 °C–200 °C at 3 °C/min or in two stages of 140 °C–180 °C at 4 °C/min and 180 °C–210 °C at 2 °C/min. The temperature of the injector and detector was 260 °C. The linear response of the detector was tested periodically with standard mixtures. Typically, two internal standards with different molecular weight (13:0 and 23:0 or 27:0) were used. Peaks were integrated with an integrator D-2500 (Hitachi Ltd., Tokyo, Japan) and identified by comparison of retention times with standards. When necessary, the identification may be confirmed by mass spectrometry (Hewlett-Packard detector model 5970B) at an ionization potential of 70 eV.

### 2.10. Statistical Analysis

Statistical analysis was performed with the GraphPad Prism Version 6.01 software (San Diego, CA, USA). In vitro experimental analyses were performed using ANOVA test followed by Bonferroni’s multiple comparison post-hoc test. To analyze the effect of EAE treatment on clinical signs and weight, day-by-day comparison was performed between treated and control animals using the Kruskal-Wallis and ANOVA test, respectively. Data are expressed as the mean ± standard error of the mean (SEM) unless otherwise stated. Statistical significance was set at *p* < 0.05.

## 3. Results

### 3.1. DHA Increased BV-2 Cells Viability

**Figure 1:**
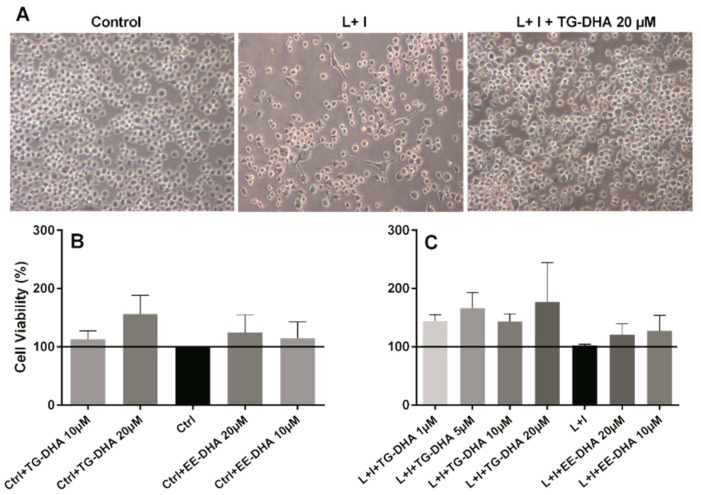
Effect of docosahexaenoic acid (DHA) on BV-2 microglial cells viability. (**A**) Optical microscopy pictures of BV-2 microglia cells cultures activated with L + I and treated with triglyceride (TG)-DHA 20 µM (right) or L + I activated but untreated (center). Controls not activated and untreated are presented in the left picture. BV-2 cells were treated with DHA and then stimulated (**C**) or not (**B**) with lipopolysaccharide + Interferon-gamma (L + I) for 24 h. Cell viability was examined by 3-(4,5-dimethyl-2-thiazolyl)-2,5-diphenyl-2H-tetrazolium bromide (MTT) reduction assays and the results were expressed as percentage of surviving cells over untreated control cells (Ctrl). Each value indicates the mean ± standard error of mean (SEM) values obtained in each of the three independent experiments. DHA was added in the triglyceride form (TG-DHA) or in the ethyl ester form (EE-DHA). In spite of the tendencies observed in favor of TG-DHA, differences were not statistically significant.

Usually DHA treatment has been associated with induction of apoptosis or cell death by other mechanisms in many cells [36,37]. However, BV-2 microglia cells treated in vitro with TG-DHA showed an increase in viability, both in basal activity (Figure 1B) and when activated with lipopolysaccharide + IFN-γ (L + I) (Figure 1A,C), with the highest increase at DHA 20 µM (Figure 1B,C), but with similar results in cells treated with 1, 5, or 10 µM (Figure 1C). These increases, however, were not statistically significant. DHA in the ethyl ester form (EE-DHA) also slightly increased BV-2 microglial cells viability in both conditions but to a lesser extent than TG-DHA.

### 3.2. DHA Attenuated NO Production and Suppressed Induction of Inflammatory Cytokines in LPS-Stimulated BV-2 Microglia Cells

It has been described that nitric oxide (NO), depending of the concentrations and pathophysiological conditions, is able to regulate cell proliferation, cell cycle arrest, and apoptosis in an opposite way. Thus, at relatively low concentrations NO may induce cell proliferation and anti-apoptotic activity, but at higher concentrations NO seems to activate pathways which induce cell cycle arrest, mitochondria respiration, senescence, or apoptosis [36,37,38].

**Figure 2:**
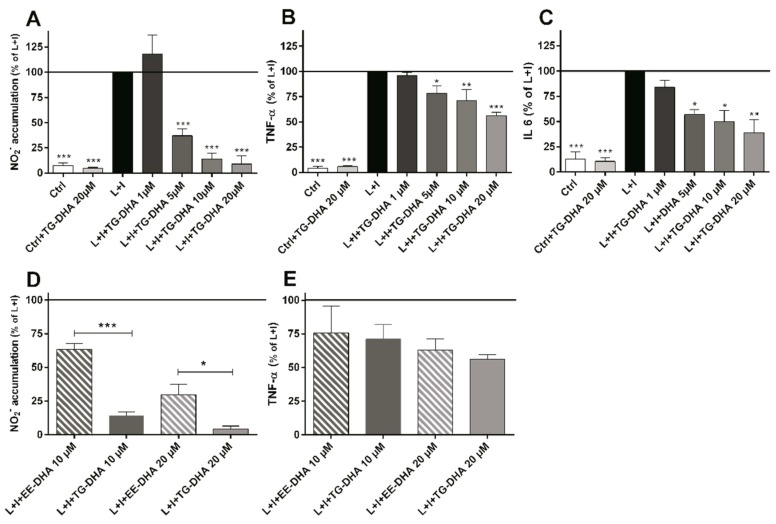
Treatment with DHA protected LPS + IFN-γ L + I-activated BV-2 microglia cells. Cells were treated with TG-DHA before stimulation with L+I. Culture supernatants of BV-2 cells were collected 24 h after L + I stimulation and assayed for nitrites by the Griess reaction (**A**) or TNF-α (**B**) or IL-6 (**C**) by Enzyme-Linked Immuno Sorbent Assay (ELISA). The effect of TG-DHA on BV-2 cells was compared with EE-DHA (10–20 µM) and assayed for nitrites (**D**) or TNF-α (**E**) under the same conditions described above. All experiments were performed in triplicate and repeated at least three times. Control wells contained the same final concentration of vehicle as compound-containing wells. * = *p* < 0.05; ** = *p* <0.01; *** = *p* < 0.005.

At doses from 5 to 20 µM, TG-DHA dramatically reduced NO production of L + I activated BV-2 microglia cells in a concentration-dependent manner (Figure 2A). Interestingly, TG-DHA at 1 µM increased, although not significantly, NO production with respect to controls, suggesting a threshold mechanism.

The effect of TG-DHA in the ability to inhibit NO production was significantly higher as compared with EE-DHA (Figure 2D).

TG-DHA also induced a significant inhibition of the production of inflammation-associated cytokines (TNF-α and IL-6) by L + I-activated BV-2 microglia cells at concentrations ranging from 1 to 20 µM (Figure 2B,C) but differences between TG-DHA and EE-DHA were not observed (Figure 2E). Taking into account the increase of viability of the BV-2 microglia cells observed above (Figure 1), the inhibition of cytokine production should be considered even higher.

### 3.3. Effects of DHA on Splenocyte Viability and Proliferation after Lymphocyte Stimulation

**Figure 3:**
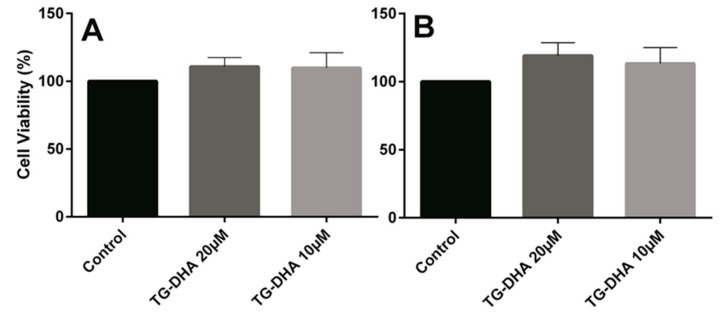
Effects of DHA in splenocyte and CD4+ T cells viability. Splenocytes (**A**) and CD4+ T cells (**B**) isolated from spleens of mice fed with a control diet were cultured ex vivo in the presence of DHA. Cell viability was examined by MTT reduction assays and the results were expressed as percentage of surviving cells as compared to control cells (Control). Each value indicates the mean ± SEM of results obtained in three independent experiments. In spite of the tendencies observed in favor of TG-DHA, no statically significant differences were found.

Mice splenocytes or purified CD4+ T cells were isolated and cultured in the presence or absence of TG-DHA. No toxicity was observed on splenocytes or CD4+ T cells. By contrast, at the doses of TG-DHA tested (1–20 µM), the viability of splenocytes was slightly higher (but not statistically significant) (Figure 3).

**Figure 4:**
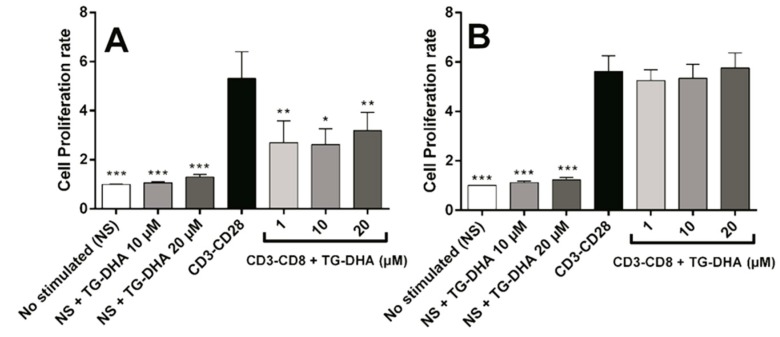
Effect of DHA on splenocytes and CD4+ T cells proliferation after anti-CD3/anti-CD28 lymphocyte stimulation. Splenocytes (**A**) and purified CD4+ T-cells (**B**) were isolated from mice and cultured in presence or absence of TG-DHA (1–20 µM). Proliferation assay was carried out after 72 h and determined using a commercial colorimetric bromodeoxyuridine (BrdU) cell proliferation kit. Proliferation rate was calculated as the absorbance of cells in the presence of anti-CD3-CD28 divided by the absorbance of cells in the absence of antibodies. * = *p* < 0.05; ** = *p* < 0.01; *** *= p* < 0.001.

When splenocytes or CD4+ T cells were induced to proliferate by culture in anti-CD3/anti-CD28 coated plates, at all doses tested, TG-DHA inhibited significantly anti-CD3/anti-CD28-stimulated splenocyte proliferation (Figure 4A). However, none of the doses tested affected proliferation of isolated CD4+ lymphocytes stimulated by anti-CD3/anti-CD28 (Figure 4B), suggesting a different regulatory mechanism [39,40].

### 3.4. Effect DHA Dietary Supplementation on Fatty Acid Profile in Mice Tissues

No significant differences were observed in plasma and red blood cell (RBC) membrane (Table 1) with respect to the profile and level of saturated, monounsaturated, polyunsaturated, omega-6, or omega-3 fatty acids in the healthy untreated and the EAE untreated groups. This demonstrated that the fatty acid profile is not dependent of the healthy status of the animals, but the diet they are fed.

**Figure 5:**
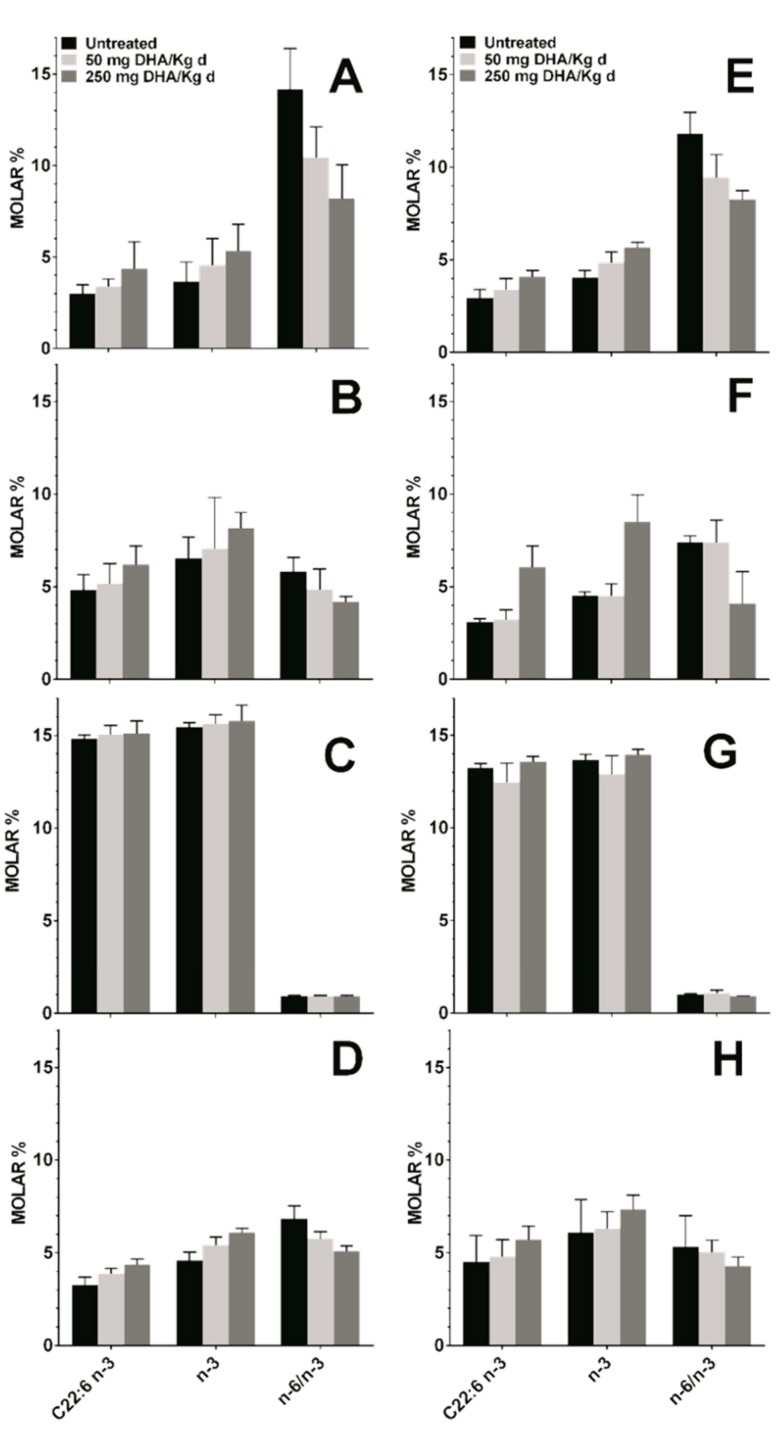
Concentration in molar % of different fatty acid in plasma (**A**,**E**), RBC membrane (**B**,**F**), brain (**C**,**G**), and spleen (**D**,**H**) in healthy mice (**A**,**B**,**C**,**D**) and in the EAE mice model (**E**,**F**,**G**,**H**) untreated (black bars) and treated with DHA at 50 (light gray bars) or 250 mg/kg/day (dark grey bars). C22:6 *n*-3, DHA; *n*-3, omega-3 fatty acids; *n*-6/*n*-3, omega-6 to omega-3 fatty acid ratio.

Mice fed orally with TG-DHA at 50 or 250 mg/kg/day for 56 days did not show any toxic effect. In the healthy and EAE groups, these treatments showed a logical upward trend in the level of DHA and omega-3 fatty acids, and a decrease in the omega-6 to omega-3 fatty acid ratio in plasma, RBC membrane, and spleen as compared to untreated animals in both the healthy group (Figure 5A,B,D) and in the EAE model (Figure 5E,F,H). The observed differences were not statistically significant, but logical due to diet, in treated with respect to untreated animals. However, in both groups, a null increase in the level of DHA or omega-3 fatty acids in the brain tissue regardless of the dose was observed (Figure 5C,G), possibly because it is a more omega-3 saturated tissue. Between-group differences were not statistically significant, which means that, with little variations, the bioavailability of TG-DHA was the same in all groups despite the health status.

### 3.5. Dietary DHA Showed a Beneficial Effect on EAE Clinical Course

To assess the effects of essential fatty acids on the clinical course of the EAE mice model, animals were treated with oral TG-DHA 50 or 250 mg/kg/day (*n* = 10 for each group) during 15 days before induction of encephalomyelitis and over the next 41 days (total days of treatment 56). The EAE-untreated group were animals without TG-DHA treatment. EAE disease severity scoring and weight measurement were performed daily.

**Figure 6:**
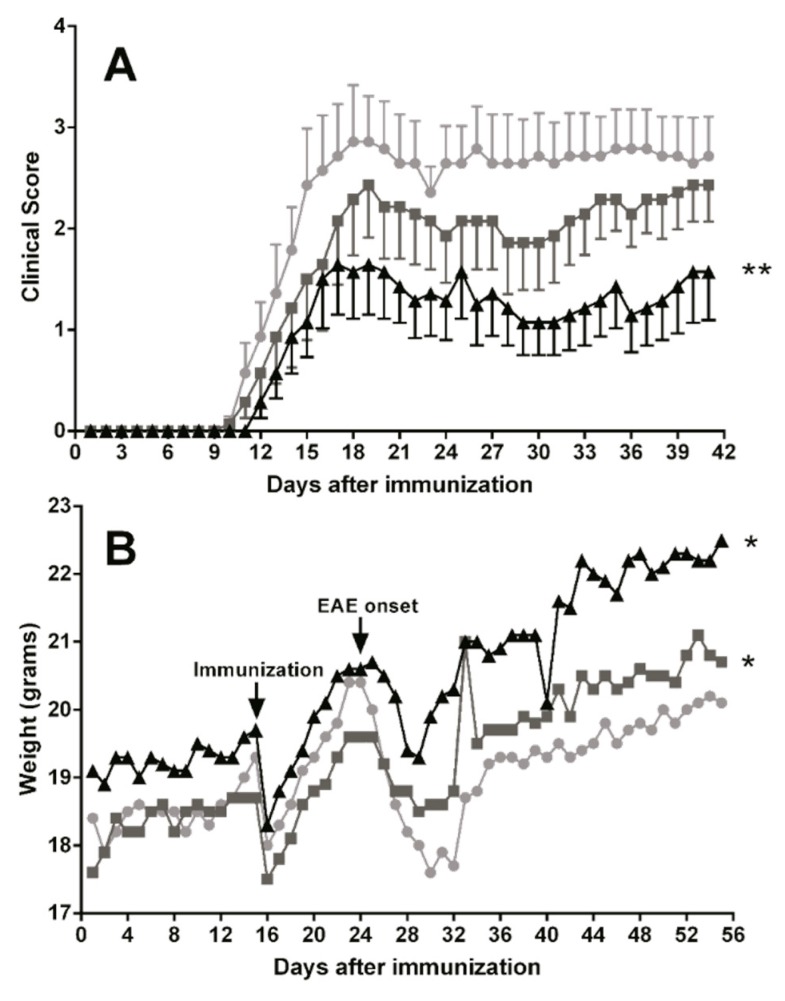
Dietary DHA improved the outcome of experimental autoimmune encephalomyelitis. EAE-induced C57BL/6 mice were fed with a normal diet in an untreated group (*n* = 10, ●) or a normal diet supplemented with TG-DHA 50 (*n* = 10, ■) or 250 mg/kg/day (*n* = 10, ▲) for 56 days. Mice were immunized with MOG35–55 as described in the Methods section. Kinetics of mean clinical score (**A**) and percentage change in weight (**B**) were controlled daily and compared day-by-day with the untreated group. Survival at the end of the observation period was also assessed. * = *p* < 0.05, ** = *p* < 0.01.

Oral TG-DHA treatment ameliorated EAE course and severity (Figure 6A) as compared to EAE-untreated animals, and especially at 250 mg/kg/day the effect was highly significant from day 15 of treatment (Figure 6A). In the same way, EAE mice treated with TG-DHA versus EAE-untreated animals also showed a better weight profile from day 30 of treatment (Figure 6B), a symptom associated with a better course of the disease. This effect was mainly observed with the 250 mg/kg/day dose (Figure 6B).

No adverse effects associated with the treatment were observed. Moreover, DHA-treated animals showed not only amelioration in the EAE neurological scoring results, but also a better aspect and lower external signs of discomfort. At the end of the study, three mice had died in the EAE-untreated group and only one in the EAE-DHA- treated group.

## 4. Discussion

Neuroinflammatory processes due to a variety of causes ranging from infectious diseases to chemical toxicity injuries are the underlying pathophysiological mechanism of many neurodegenerative disorders in which microglia cells play an important role. In this study, the triglyceride of omega-3 polyunsaturated fatty acid docosahexaenoic acid (TG-DHA) has been assayed in vitro and in vivo as compared to the DHA ethyl ester form, to determine its capacity to protect cells from oxidative stress injury and inflammatory cytokine damage activity. The beneficial effects of TG-DHA have also been studied in a mice model of EAE.

The role of omega-3 polyunsaturated fatty acid (*n*-3 PUFA) in different cellular systems is still controversial, ranging from death induction by means of oxidative stress products [36,37,41] to protective activity increasing survival and anti-oxidative stress [42,43,44,45,46]. However, the anti-inflammatory activity of omega-3 PUFA and especially of DHA has been reported for a long time as a general behavior in dendritic [26,30,47,48,49] and microglia cells [11,27,28,29]. Moreover, treatment with DHA has been reported to ameliorate neurodegenerative diseases involving neuroinflammation [30,31], including EAE [3]. In the present study, we demonstrated that with respect to EE-DHA, TG-DHA treatment shows an increased protection on BV-2 microglia cells activated with LPS and IFN-γ from toxicity, increasing viability, attenuating NO production, and suppressing the induction of pro-inflammatory cytokines (Figure 1 and Figure 2). This behavior could be related to the better bioavailability of the TG-DHA compared to EE-DHA [32,33].

TG-DHA significantly inhibited mice splenocyte proliferation but did not affect proliferation of isolated CD4+ lymphocytes cultured in anti-CD3/anti-CD28 coated plates (Figure 4). This fact suggests the possibility of an indirect mechanism in which the anti-proliferative effects of TG-DHA could be associated with a modulation of the monocyte-dendritic cell antigen presentation activity, as observed previously [26]. However, T-cell inhibition has been confirmed by other authors [39,40], suggesting that anti-proliferative effects of TG-DHA are probably associated with a macrophage/monocyte modulation instead of a direct activity on lymphocytic immunomodulation. Although molecular mechanisms are not exclusive of the myeloid cells and omega-3 fatty acids could inhibit T-lymphocytes through several mechanisms (like PPARγ upregulation and activation which induce Treg proliferation) [25,50,51], at the conditions of the present assay, there probably was not enough time to develop regulatory T-cells to induce CD4+ T-cell inhibition (Figure 4B).

No differences in the profile and level of fatty acids from plasma and RBC membranes of healthy animals were observed in comparison with those from the mouse EAE model (Table 1). When mice were fed with a supplement of 50 to 250 mg/kg/day of TG-DHA, a slightly but upward trend of the level of DHA and the omega-3 in the peripheral tissues (plasma, RBC, and spleen) was observed in a dose-dependent manner, both in healthy animals and in those with a EAE (Figure 5). However, no changes or almost undetectable changes were observed in the CNS tissues (Figure 5).

In a mouse model of EAE, when comparing untreated animals with high and low doses of TG-DHA treatment, it was observed that high doses significantly ameliorated the clinical course and severity of disease in a dose and time dependent manner (Figure 6), in relation to both the EAE clinical score and weight profile (a symptom associated with a better course of the disease). Our results with TG-DHA confirm and improve previous results from Kong W. et al. [3], demonstrating that TG-DHA with a DHA high content (>70% of total fatty acids) is a more effective treatment and improves the outcome and disease evolution in the mouse model of EAE. Our data suggest that prophylactic administration of TG-DHA confers significant protection against the development of EAE at clinical and molecular levels.

Among the strengths of this study were the approach of the problem with the types of cells involved in the neuroinflammation processes and their phenotypic and functional changes, the comparative study of different doses of DHA in its different chemical forms and with a high content in DHA, the strict control of the animals involved in the study, and the assessment of the variations in the composition of the content of fatty acids in cell membranes and plasma. However, some limitations in this work remain. Further, there is a paucity of studies on the mechanisms of DHA on neuroinflammation pathways in vivo. It is hypothesized that omega-3 PUFA are anti-inflammatory via their enzymatically-derived metabolites, however, comprehensive lipidomics profiling during neuroinflammation has not been reported yet and the pathways that they mediate should be investigated. Nevertheless, the results presented here advance our understanding of the anti-inflammatory effect of DHA in EAE, and further studies should be conducted to address the concerns raised.

## 5. Conclusions

We conclude that DHA, a natural product that can be taken as a dietary supplement, showed no toxic effects on microglial cells in the in vitro studies as well as the in vivo studies of mice treated with high DHA doses for a long period of time. In the supplement used in this study, DHA was present in the form of triglycerides, nutrients naturally recognized by the body, to guarantee high digestibility and intestinal absorption. Furthermore, TG-DHA modulates the in vitro activity of microglia cells and T-cells, suggesting a neuronal cell protection from cytotoxic insults. In the EAE mouse model, treatment with TG-DHA had a beneficial effect on the clinical course and severity of autoimmune encephalomyelitis. The beneficial and protective effects of TG-DHA are mediated, at least in part, through oxidant effects and by modulating the autoimmune responses within the CNS as well as at the systemic level. According to these results, TG-DHA may be a promising nutritional immunomodulating agent in neuroinflammatory processes, suggesting potential favorable effects in human neurodegenerative disorders.

## Figures and Tables

**Table 1:** Fatty acid composition of total lipids from plasma and red blood cell (RBC) membrane isolated from healthy untreated (Ctl) animals or with autoimmune encephalomyelitis (EAE)-induced disease.

% of Total Fatty Acids				
Total Fatty Acids	Plasma		RBC	
	Ctl (*n* = 7)	EAE (*n* = 7)	Ctl (*n* = 7)	EAE (*n* = 7)
SFA	31.70 ± 0.96	31.70 ± 0.49	47.53 ± 4.23	50.25 ± 6.89
MUFA	16.97 ± 1.19	16.23 ± 1.35	13.27 ± 0.57	14.54 ± 0.81
PUFA	51.33 ± 1.12	52.07 ± 1.36	39.20 ± 4.74	35.21 ± 7.60
PUFA *n*-6	47.68 ± 1.36	47.60 ± 1.24	32.01 ± 4.43	29.93 ± 5.75
PUFA *n*-3	3.65 ± 1.08	3.87 ± 0.91	6.55 ± 1.12	5.28 ± 1.93
*n*-6/*n*-3 ratio	14.17 ± 2.22	11.07 ± 2.25	5.83 ± 0.74	6.09 ± 1.29
C22:6 *n*-3	3.01 ± 0.48	2.96 ± 0.79	4.83 ± 0.83	3.39 ± 1.08

Data are presented as mean ± standard deviation (SD). SFA: saturated fatty acid; MUFA: monounsaturated fatty acid; PUFA: polyunsaturated fatty acids; *n*-6: omega-6; *n*-3: omega-3; C22:6 omega-3: DHA.
